# Analysis of risk factors for lymph node metastasis and prognosis study in patients with early gastric cancer: A SEER data-based study

**DOI:** 10.3389/fonc.2023.1062142

**Published:** 2023-03-17

**Authors:** Jinzhou Li, Ting Cui, Zeping Huang, Yanxi Mu, Yalong Yao, Wei Xu, Kang Chen, Haipeng Liu, Wenjie Wang, Xiao Chen

**Affiliations:** ^1^The Second Clinical Medical College, Lanzhou University, Lanzhou, China; ^2^Department of General Surgery, The Second Hospital of Lanzhou University, Lanzhou, China

**Keywords:** early gastric cancer, lymph node metastasis, prognosis, nomogram, SEER

## Abstract

**Background:**

Lymph node status is an important factor in determining the prognosis of patients with early gastric cancer (EGC) and preoperative diagnosis of lymph node metastasis (LNM) has some limitations. This study explored the risk factors and independent prognostic factors of LNM in EGC patients and constructed a clinical prediction model to predict LNM.

**Methods:**

Clinicopathological data of EGC patients was collected from the public Surveillance, Epidemiology, and End Results (SEER) database. Univariate and multivariate logistic regression was used to identify risk factors for LNM in EGC patients. The performance of the LNM model was evaluated by C-index, calibration curve, receiver operating characteristic (ROC) curve, decision curve analysis (DCA) curve, and clinical impact curve (CIC) based on the results of multivariate regression to develop a nomogram. An independent data set was obtained from China for external validation. The Kaplan-Meier method and Cox regression model were used to identify potential prognostic factors for overall survival (OS) in EGC patients.

**Results:**

A total of 3993 EGC patients were randomly allocated to a training cohort (n=2797) and a validation cohort (n=1196). An external cohort of 106 patients from the Second Hospital of Lanzhou University was used for external validation. Univariate and multivariate logistic regression showed that age, tumor size, differentiation, and examined lymph nodes count (ELNC) were independent risk factors for LNM. Nomogram for predicting LNM in EGC patients was developed and validated. The predictive model had a good discriminatory performance with a concordance index (C-index) of 0.702 (95% CI: 0.679-0.725). The calibration plots showed that the predicted LNM probabilities were the same as the actual observations in both the internal validation cohort and external validation cohort. The AUC values for the training cohort, internal validation cohort and external validation cohort were 0.702 (95% CI: 0.679-0.725), 0.709 (95% CI: 0.674-0.744) and 0.750(95% CI: 0.607-0.892), respectively, and the DCA curves and CIC showed good clinical applicability. The Cox regression model identified age, sex, race, primary site, size, pathological type, LNM, distant metastasis, and ELNC were prognostic factors for OS in EGC patients, while a year at diagnosis, grade, marital status, radiotherapy, and chemotherapy were not independent prognostic factors.

**Conclusion:**

In this study, we identified risk factors and independent prognostic factors for the development of LNM in EGC patients, and developed a relatively accurate model to predict the development of LNM in EGC patients.

## 1 Introduction

Gastric cancer remains important cancer worldwide and is responsible for over one million new cases in 2020 and an estimated 769,000 deaths, ranking fifth for incidence and fourth for mortality globally (1). In recent years, the diagnosis of early gastric cancer (EGC) has rapidly increased due to improvements in universal screening and endoscopic techniques. According to the staging manual jointly developed by the American Joint Committee on Cancer (AJCC) and the International Union Against Cancer (UICC), EGC is defined as a superficial gastric lesion confined to the mucosa (T1a) and submucosa (T1b), regardless of the lymph node status (2). EGC accounts for more than 50% of total cases in Japan and Korea, whereas in Western countries, it accounts for only about 20% (3).

In general, endoscopic resections, such as endoscopic mucosal resection (EMR) and endoscopic submucosal dissection (ESD), can be performed when the likelihood of lymph node metastasis (LNM) is minimal and the size and location of the lesion allow for whole-block resection (4). Lesions are considered absolute indications for endoscopic therapy if they are presupposed to have a <1% risk of LNM (5). EGC progresses slowly, but approximately 20% of patients with EGC develop LNM (6). LNM is an independent risk factor affecting the prognosis of patients with EGC and determining the extent of lymph node dissection (7). The 2018 edition of the Chinese guidelines for the management of gastric cancer states that gastrectomy combined with lymph node dissection remains the primary treatment for patients with EGC with LNM (8). However, in some cases, the occurrence of LNM in EGC patients cannot be identified, resulting in receiving unreasonable endoscopic treatment. For patients with EGC who develop distant metastases, the Japanese guidelines for gastric cancer recommend systemic therapy (9). However, due to the limited number of cases, the main risk factors and prognostic factors for LNM in EGC patients have not been well studied. Therefore, the prediction of the risk of LNM in EGC and the identification of prognostic factors are important prerequisites and bases to guide the rational clinical selection of treatment modalities and improve survival.

To date, several studies have identified some clinicopathological features of EGC as risk factors for predicting LNM, such as age, tumor size, lymphatic invasion, depth of invasion, grade, and intestinal type associated with LNM (10–13), and corresponding predictive models, including nomograms and scoring systems, have been developed to provide evidence for clinical decision-making, but there is still no consensus on their applicability to the clinic.

The nomogram is widely used for cancer prognosis, mainly because of its ability to reduce statistical prediction models to single-digit estimates of the probability of an event (death or recurrence) (14). Therefore, a nomogram for preoperative assessment of the risk of LNM in EGC can help clinicians choose appropriate treatment modalities. Cox regression analysis can identify risk factors associated with prognosis and may help patients and physicians in various aspects of decision-making.

In this study, using the SEER database, the clinicopathological characteristics of EGC patients with and without LNM were first compared. Logistic regression analysis was then used to identify risk factors associated with LNM. A Cox proportional hazards model was further employed to identify risk factors associated with the prognosis of EGC patients. The results of this population-based study will help improve the management of patients with EGC.

## 2 Methods

### 2.1 Ethics approval and consent to participate

The study was a retrospective study based on the SEER database. The authors obtained authorization to exact and analyze the research data stored in the SEER program from the National Cancer Institute, USA (reference number 19369-Nov2021). All procedures followed were in accordance with the Declaration of Helsinki and subsequent versions, and were approved by the Ethics Committee of the Second Hospital of Lanzhou University (approval number: 2022A-623).

### 2.2 Data sources and population selection

Clinical data of EGC patients in the SEER database were collected using SEER*Stat software (version 8.3.9; www.seer.cancer.gov) and using personal ID (account number: 12145-Nov2020). Since the SEER database is public, informed consent is not required, therefore, this study was exempted from review by the ethics committee of our institution (15).

**Figure 1:**
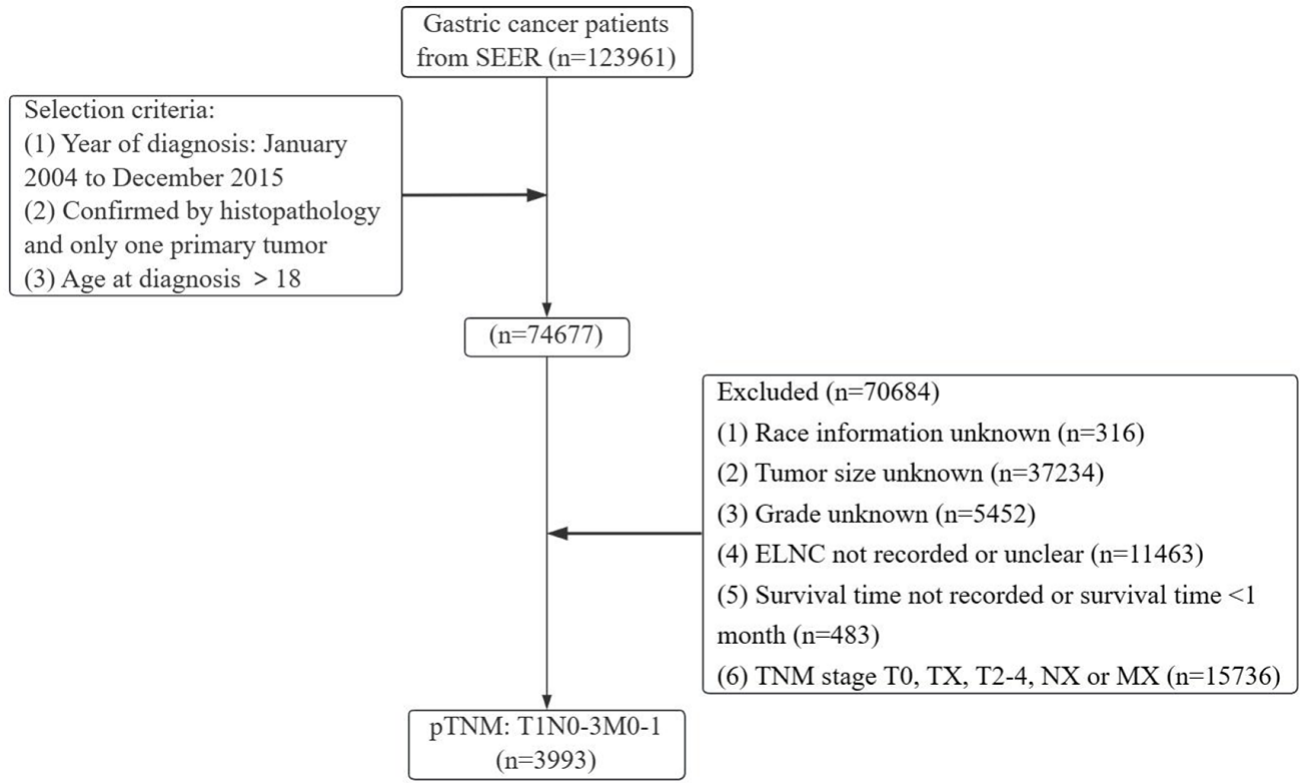
Flow chart of the patient screening process in the Surveillance, Epidemiology, and End Results.

**Figure 2:**
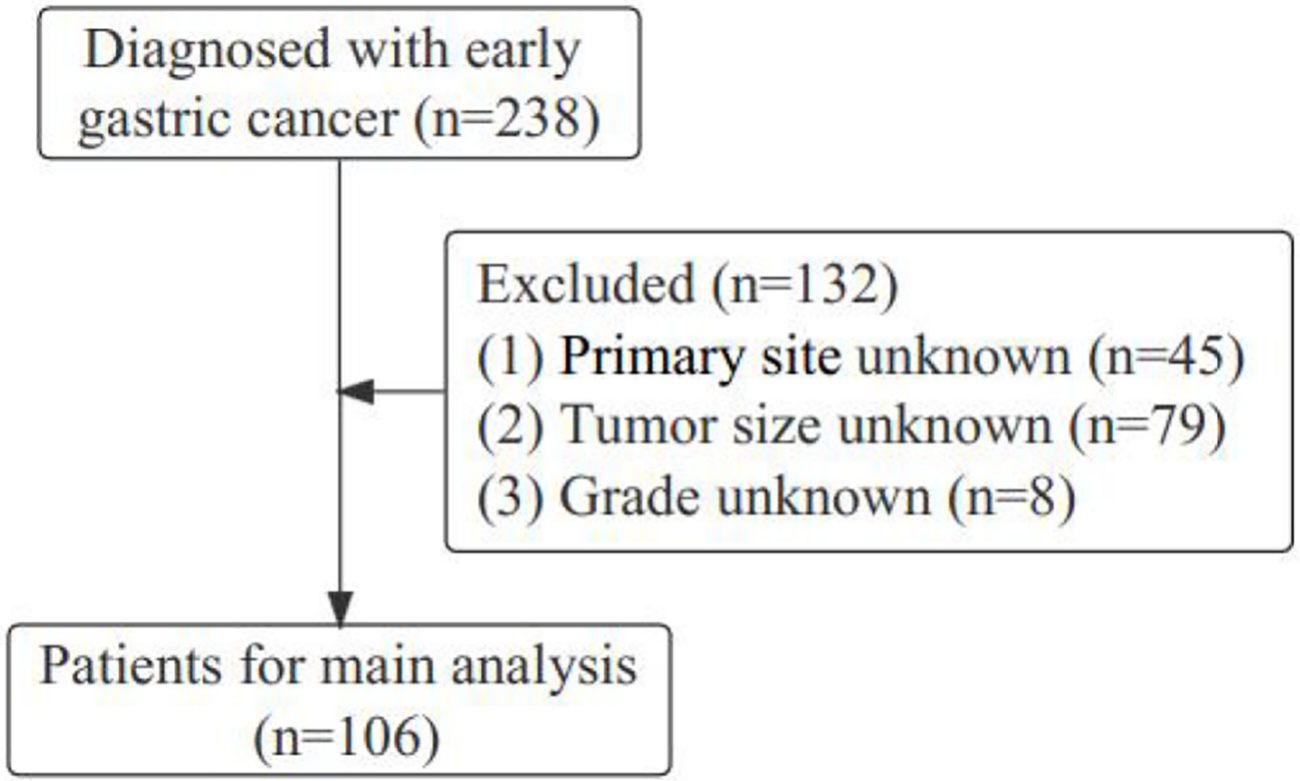
The diagram of the patient screening process in the Second Hospital of Lanzhou University.

Gastric cancer (C16.0-16.9) patients were identified from the SEER database according to the website recoding classification. And 106 patients from the Second Hospital of Lanzhou University who underwent gastric surgery from January 2015 to December 2017. The inclusion criteria used in this study were as follows: (1) Year of diagnosis: January 2004 to December 2015; (2) Histopathologically confirmed and only one primary tumor was gastric cancer; (3) Age > 18 years old; (4) The postoperative pathological stage was: T1N0-3M0-1. Exclusion criteria: (1) unknown ethnicity; (2) unknown tumor size; (3) unknown degree of differentiation; (4) ELNC was not recorded or unclear; (5) survival time was not recorded or survival time after diagnosis was less than 1 month. The process of patient screening is shown in **Figure 1** and **Figure 2**. Patients from the SEER database were randomized into a training cohort and an internal validation cohort. The training cohort included 2,797 patients, while the internal validation cohort included 1,196 patients and 106 patients (the Second Hospital of Lanzhou University). The primary clinical endpoint was OS.

This study was based on public data from the SEER database without interacting with human subjects or using personal identifying information. This research was therefore exempted from review by the Human Subjects Committee of Institutional Review Board of the Second Hospital of Lanzhou University.

### 2.3 Variables and outcomes

The following variables were collected: year at diagnosis (2004-2007, 2008-2011, 2012-2015), age (≤70 years, >70 years), sex, marital status (married, other), race (white, black, other races), tumor size (1-10mm, 11-20mm, 21-30mm, >30mm). Histological types were classified using the coding pattern of the ICD-O-3 (International Classification of Diseases in Oncology, Third Edition) (16), adenocarcinomas (8140, 8143-8144, 8210-8211, 8255, 8260-8263, 8310, 8323, 8480-8481, 8574, 8576), signet ring cell carcinoma (8490) and others. Grade (grade I: well differentiated, grade II: moderately differentiated, grade III: poorly differentiated, grade IV: undifferentiated), primary site (cardia: C16.0-cardia; non-cardia: C16.1-stomach bottom, C16.2-body, C16.3-gastric antrum, C16.4-pylorus, C16.5-lesser curvature, C16.6-lesser curvature, C16.8-overlapping lesions of the stomach). The optimal cut-off value of the ELNC by X-tile software (≤11, >11), and the TNM staging were restaged according to the 8th edition of the AJCC/UICC guidelines (N0, non-N0: N1, N2, N3). Treatment-related included radiotherapy (yes, no/unknown), chemotherapy (yes, no/unknown), and surgery (yes, no/unknown). Overall survival (OS) was interpreted as the period from the date of diagnosis to the last follow-up or death from any cause.

### 2.4 Statistical methods

The patients in the database were randomly divided into the training cohort and validation cohort in a ratio of 7:3. The training cohort is used for model development, and the validation cohort is used for evaluation and validation. The optimal cutoff value of ELNC associated with LNM was calculated using X-tile software. The basic characteristics of the included patients were described by number and percentage (n, %). Each variable’s contribution in predicting LNM of EGC in the training cohort was tested by univariate logistic analysis. Variables that were statistically significant were further analyzed by multivariate logistic regression. The odds ratios (OR) with corresponding 95% confidence interval (CI) was calculated. Risk factors which were statistically significant in the multivariate analysis were used to construct a predictive nomogram to predict the LNM. Nomogram performance was evaluated with respect to discrimination and calibration. For discrimination ability, the nomogram was evaluated using the area under the receiver operating characteristic (ROC) curve (AUC). Calibration curves were plotted to verify the accuracy and reliability of the nomogram. Internal and external validations were performed to validate the nomogram. Moreover, decision curve analysis (DCA) was plotted to measure the applicability of the nomogram to clinical practice. After exploring the risk factors of LNM in EGC patients, we also used the Kaplan-Meier method and Cox regression model to analyze the prognosis of EGC patients. All statistical analyses were performed with v SPSS 25.0 and R software v4.0.3 (https://www.r-project.org/). Two-sided p-values were considered statistically significant.

## 3 Results

### 3.1 Patient baseline characteristics

According to the inclusion and exclusion criteria, a total of 3993 EGC patients were identified, with an overall LNM rate of 20.84% and an overall distant metastasis rate of 1.53%. The R language random number method was used to divide the research subjects according to the ratio of 7:3, including 2797 cases in the training cohort and 1196 cases in the validation cohort. The optimal cutoff value of ELNC associated with LNM was calculated using X-tile software as 11. The demographic and clinicopathological characteristics of the training cohort and the validation cohort are shown in **Table 1**. The two groups of patients were diagnosed in a year, age, sex, race, pathological type, primary site, tumor size, grade, marital status, ELNC, and LNM. There was no significant difference between radiotherapy and chemotherapy (P>0.05). The overall division of the two groups conformed to simple randomization and was comparable.

**Table 1:** Demographic and clinicopathologic variables in the training and validation cohort.

Variables	All patients	Training cohort, n (%)	Validation cohort, n (%)	*χ*^2^	*p* value
Total	3993	2797	1196		
Year of diagnosis				0.139	0.933
2004-2007	1271	887(31.70)	384(32.10)		
2008-2011	1390	972(34.70)	418(34.90)		
2012-2015	1332	938(33.60)	394(32.90)		
Age at diagnosis				0.088	0.767
≤70	2069	1445(51.70)	624(52.20)		
>70	1924	1352(48.30)	572(47.80)		
Gender				0.002	0.962
Male	2445	1712(61.20)	733(61.30)		
Female	1548	1085(38.80)	463(38.70)		
Race				0.753	0.686
White	2528	1781(63.70)	747(62.50)		
Black	466	327(11.70)	139(11.60)		
Other	999	689(24.60)	310(25.90)		
Primary site				0.442	0.506
Cardia	1113	771(27.60)	342(28.60)		
Non-cardia	2880	2026(72.40)	854(71.40)		
Tumor size (mm)				5.927	0.115
1-10	946	652(23.30)	294(24.60)		
11-20	1201	870(31.10)	331(27.70)		
21-30	824	580(20.70)	244(20.40)		
>30	1022	695(24.80)	327(27.30)		
Histology				0.008	0.996
Adenocarcinoma	3125	2188(78.20)	937(78.30)		
SRC	636	446(15.90)	190(15.90)		
Others	232	163(5.80)	69(5.80)		
Grade				2.622	0.454
Grade I	557	376(13.40)	181(15.10)		
Grade II	1509	1073(38.40)	436(36.50)		
Grade III	1864	1305(46.70)	559(46.70)		
Grade IV	63	43(1.50)	20(1.70)		
Marital status				1.284	0.257
Married	2246	1557(55.70)	689(57.60)		
Others	1747	1240(44.30)	507(42.40)		
ELNC				0.157	0.692
≤11	1725	1214(43.40)	511(42.70)		
>11	2268	1583(56.60)	685(57.30)		
N Stage				1.078	0.299
N0	3161	2202(78.70)	959(80.20)		
N1/N2/N3	832	595(21.30)	237(19.80)		
M Stage				0.849	0.357
M0	3932	2751(98.40)	1181(98.70)		
M1	61	46(1.60)	15(1.30)		
Chemotherapy				0.672	0.412
Yes	708	505(18.10)	203(17.00)		
None/Unknown	3285	2292(81.90)	993(83.00)		
Radiation				0.197	0.657
Yes	478	339(12.10)	139(11.60)		
None/Unknown	3515	2458(87.90)	1057(88.40)		

P value was calculated by χ2 test; EGC: early gastric cancer; SRC, signet ring cell carcinoma; ELNC, examined lymph node count.

As for the external validation cohort, 106 patients from our center were included in this study. In the external validation cohort, the mean value of age was 57.6 ± 8.6, the mean value of tumor size was 2.1 ± 1.3 cm, and the average number of ELNC removed during surgery was 17.7 ± 8.7. Continuous variables were converted to categoric variables. The clinicopathological characteristics in the Second Hospital of Lanzhou University are listed in **Table 2**.

**Table 2:** The clinicopathological characteristics in the Second Hospital of Lanzhou University.

Variables	No. of patients (%), n=106
Age at diagnosis	
≤70	97(91.5%)
>70	9(8.5%)
Gender	
Male	72(67.9)
Female	34(32.1)
Primary site	
Cardia	10(9.4)
Non-cardia	96(90.6)
Tumor size (mm)	
1-10	30(28.3)
11-20	38(35.8)
21-30	23(21.7)
>30	15(14.2)
Grade	
Grade I	20(18.9)
Grade II	60(56.6)
Grade III	24(22.6)
Grade IV	2(1.9)
ELNC	
≤11	24(22.6)
>11	82(77.4)
N Stage	
N0	92(86.8)
N1/N2/N3	14(13.2)

### 3.2 Analysis of risk factors for LNM in EGC patients

#### 3.2.1 Univariate logistic regression analysis

To identify risk factors for LNM in EGC patients. We performed univariate logistic regression and multivariate logistic regression to adjust for confounders. Univariate logistic regression results (**Table 3**) showed that age, tumor size, grade, histology, and ELNC were related to LNM.

**Table 3:** Univariate and multivariate logistic regression analysis to identify risk factors for LNM in EGC patients.

Variable	Univariate analysis		Multivariate analysis	
	OR (95% CI)	*P* value	OR (95% CI)	*P* value
Year of diagnosis				
2004-2007	Ref			
2008-2011	0.884(0.709-1.102)	0.272		
2012-2015	0.856(0.685-1.07)	0.173		
Age at diagnosis				
≤70	Ref		Ref	
>70	0.796(0.663-0.955)	0.014	0.847(0.698-1.026)	0.090
Gender				
Male	Ref			
Female	0.932(0.773-1.123)	0.459		
Race				
White	Ref			
Black	1.046(0.787-1.389)	0.758		
Other	0.911(0.733-1.133)	0.403		
Primary site				
Cardia	Ref			
Non-cardia	0.919(0.752-1.123)	0.409		
Tumor size (mm)				
1-10	Ref		Ref	
11-20	1.717(1.255-2.35)	0.001	1.556(1.132-2.140)	0.006
21-30	3.294(2.403-4.515)	<0.001	2.946(2.138-4.061)	<0.001
>30	4.643(3.438-6.271)	<0.001	4.258(3.135-5.782)	<0.001
Histology				
Adenocarcinoma	Ref		Ref	
SRC	1.129(0.883-1.443)	0.333	0.839(0.637-1.105)	0.211
Others	1.679(1.182-2.384)	0.004	1.182(0.810-1.726)	0.386
Grade				
Grade I	Ref		Ref	
Grade II	2.778(1.8344.206)	<0.001	2.523(1.654-3.850)	<0.001
Grade III	4.753(3.1747.116)	<0.001	4.078(2.700-6.159)	<0.001
Grade IV	3.766(1.6838.428)	0.001	2.907(1.270-6.652)	0.012
Marital status				
Married	Ref			
Others	1.046(0.8721.255)	0.628		
ELNC				
≤11	Ref		Ref	
>11	1.734(1.4342.097)	<0.001	1.586(1.310-1.934)	<0.001

#### 3.2.2 Multivariate logistic regression analysis

Factors with P<0.1 in univariate logistic regression were included in multivariate logistic regression, and four significant risk factors for LNM were finally included: age at diagnosis, tumor size, grade, and ELNC (**Table 3**). In terms of age, older patients had a lower risk of developing LNM (OR=0.847, 95%CI=0.698-1.026, P=0.090). In terms of tumor size, larger patients had an increased risk of developing LNM, 11-20mm compared with EGC patients with size 1-10mm (OR=1.556, 95%CI=1.132-2.140, P=0.006), 20-30mm (OR=2.946, 95%CI=2.138-4.061, P<0.001), greater than 30mm (OR=4.258, 95%CI=3.135-5.782, P<0.001), compared with well-differentiated EGC cancer, moderately differentiated (OR=2.523, 95%CI=1.654-3.850, P<0.001), poorly differentiated (OR=4.078, 95%CI=2.700-6.159, P<0.001) and undifferentiated (OR=2.907, 95%CI=1.270-6.652, P=0.012) patients had a higher risk of LNM. The risk of LNM increased when the ELNC was higher (OR=1.586, 95%CI=1.310-1.934, P<0.001).

#### 3.2.3 Establish a nomogram of LNM

**Figure 3:**
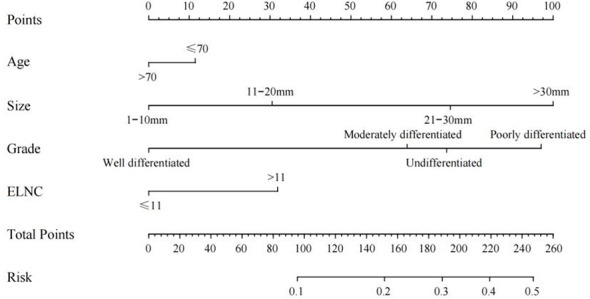
Nomogram for predicting the LNM.

Based on the results of univariate logistic regression, we established a nomogram plot including age, tumor size, grade, and ELNC for examination to predict the probability of LNM in EGC patients **(** **Figure 3**). In this model, tumor size and grade were the biggest predictors of LNM. The resampling method was used for internal validation of the nomogram model, and the ROC curve and C-Index were used to evaluate the accuracy of the model; the calibration curve was used to evaluate the consistency of the predicted value with the actual survival situation; the DCA curve and the CIC were used to evaluate the net benefit of constructing the model.

**Figure 4:**
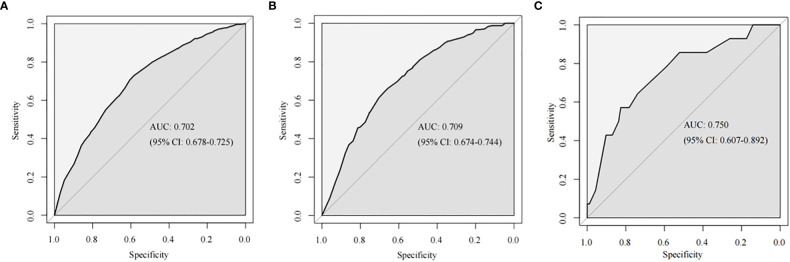
The ROC curves of the nomogram for predicting LNM in the training cohort **(A)**, internal validation cohort **(B)** and external validation **(C)**.

**Figure 5:**
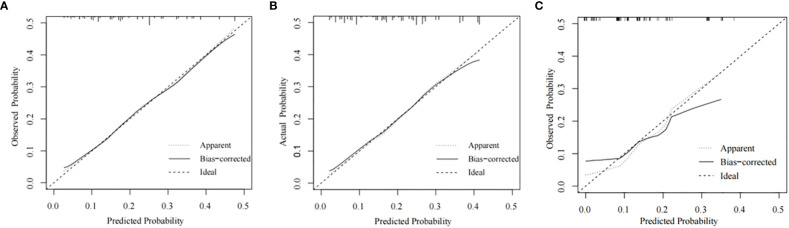
Calibration plots for the nomogram. Calibration plots for the nomogram in the training cohort **(A)**, the internal validation cohort **(B)**, and the external validation cohort **(C)**.

**Figure 6:**
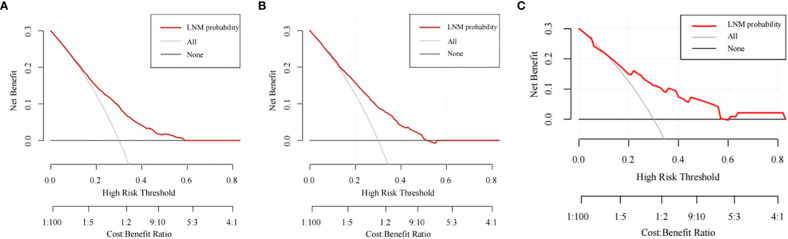
The DCA curves of the nomogram for predicting the LNM in the training cohort **(A)**, the internal validation cohort **(B)**, and the external validation cohort **(C)**.

**Figure 7:**
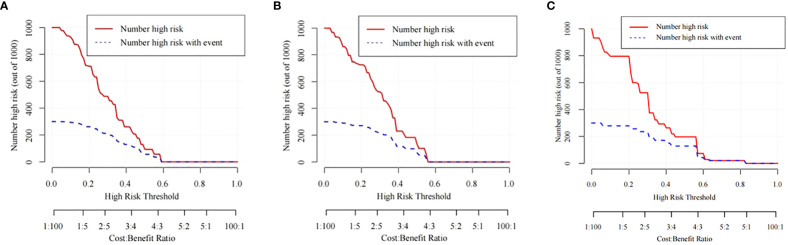
The CIC curves of the nomogram for predicting the LNM in the training cohort **(A)**, the internal validation cohort **(B)**, and the external validation cohort **(C)**.

The training cohort ROC (**Figure 4A**) showed great discrimination against the nomogram, with an AUC value of 0.702 and a model C-index of 0.702 (95% CI=0.679-0.25). The calibration curve showed high accuracy (**Figure 5A**). In addition, DCA and CIC showed that the nomogram showed a threshold probability of 0.2-0.6 with good gain (**Figures 6A** and **7A**). Also in the internal validation cohort, the AUC value of the ROC curve was 0.709 (**Figure 4B**) and the C-index was 0.709 (95% CI=0.674-0.744). The calibration curve showed high accuracy (**Figure 5B**). In addition, DCA and CIC showed that the nomogram showed a threshold probability of 0.2-0.6 with good gain (**Figures 6B** and **7B**). The evaluation effect of the external validation cohort is essentially the same as the internal validation cohort (**Figures 4C**, **5C**, **6C**, **7C**).

### 3.3 Survival analysis of EGC patients

After exploring the risk factors of LNM in EGC patients, we also used the Kaplan-Meier method and Cox regression model to analyze the prognosis of EGC patients. Risk factors related to survival in EGC patients were analyzed by Cox proportional hazards regression model. Both univariate and multivariate results showed that age, gender, race, primary site, tumor size, pathological type, LNM, ELNC, and distant metastasis were significantly associated with tumor OS, while the year of diagnosis, grade, radiotherapy, and chemotherapy were not associated with OS (**Table 4**).

**Table 4:** Cox regression of univariate and multivariate analysis associated with OS of EGC.

Variable	Univariate analysis		Multivariate analysis	
	HR (95% CI)	P value	HR (95% CI)	P value
Year of diagnosis				
2004-2007	Ref		Ref	
2008-2011	0.901(0.807-1.006)	0.063	0.973(0.870-1.087)	0.624
2012-2015	0.814(0.713-0.929)	0.002	0.900(0.787-1.029)	0.124
Age				
≤70	Ref		Ref	
>70	2.260(2.051-2.492)	<0.001	2.356(2.130-2.607)	<0.001
Gender				
Male	Ref		Ref	
Female	0.780(0.707-0.861)	<0.001	0.763(0.689-0.845)	<0.001
Race				
White	Ref		1.170(1.008-1.358)	0.039
Black	0.997(0.863-1.151)	0.964	0.784(0.691-0.890)	<0.001
Other	0.679(0.603-0.766)	<0.001	0.775(0.691-0.870)	<0.001
Primary site				
Cardia	Ref			
Non-cardia	0.820(0.741-0.908)	<0.001	0.775(0.691-0.870)	<0.001
Tumor size (mm)				
1-10	Ref			
11-20	1.175(1.024-1.348)	0.022	1.106(0.963-1.270)	0.153
21-30	1.465(1.268-1.693)	<0.001	1.242(1.073-1.438)	0.004
>30	1.563(1.363-1.793)	<0.001	1.338(1.160-1.542)	<0.001
Histology				
Adenocarcinoma	Ref		0.832(0.718-0.964)	0.014
SRC	0.660(0.572-0.761)	<0.001	0.852(0.679-1.071)	0.170
Others	0.712(0.569-0.890)	0.003		
Grade				
Grade I	Ref			
Grade II	1.080(0.934-1.250)	0.300		
Grade III	0.968(0.839-1.118)	0.658		
Grade IV	0.801(0.518-1.239)	0.319		
Marital status				
Married	Ref			
Others	1.077(0.980-1.183)	0.122		
ELNC				
≤11	Ref			
>11	0.693(0.631-0.762)	<0.001	0.717(0.650-0.790)	<0.001
N Stage				
N0	Ref			
N1/N2/N3	1.624(1.459-1.807)	<0.001	1.670(1.492-1.869)	<0.001
M Stage				
M0	Ref			
M1	2.360(1.738-3.207)	<0.001	1.914(1.401-2.615)	<0.001
Chemotherapy				
Yes	Ref			
None/Unknown	0.953(0.842-1.078)	0.445		
Radiation				
Yes	Ref			
None/Unknown	0.879(0.765-1.011)	0.072		

## 4 Discussion

EGC is defined by the Japanese Society for Gastrointestinal Endoscopy as an invasive gastric cancer that does not invade deeper than the submucosa and is not associated with LNM. Currently, only South Korea and Japan have relatively complete gastric cancer prevention and screening systems in the world (17). In recent years, with the gradual popularization and application of endoscopic EMR and ESD surgery, the clinical diagnosis and treatment of patients with EGC have developed from a simple surgical operation to a two-way choice of endoscopic resection or surgical treatment. The advantages of endoscopic resection of EGC are less trauma, quick postoperative recovery, high quality of life, and long-term efficacy comparable to surgery. According to the Japanese gastric cancer treatment guidelines, the absolute indications for endoscopic resection are differentiated adenocarcinoma of cT1a without ulceration or differentiated adenocarcinoma of cT1a with ulceration diameter ≤ 3cm. Endoscopic resection of undifferentiated cT1a carcinomas ≤ 2 cm in diameter without ulcerative manifestations is considered an expanded indication (9). However, even with strict adherence to the indications for endoscopic therapy, at least 1.9 percent of cases recur after resection of the lesion, with intervals ranging from 4 months to more than 10 years (18). One of the important risk factors for recurrence is LNM. However, the main drawback of endoscopic resection is that it cannot achieve perigastric lymph node dissection. For patients with EGC with LNM, surgery is still required to achieve radical tumor resection. Previous studies have shown that preoperative diagnosis of lymph node status still has certain limitations. Therefore, effective prediction and accurate prognostic assessment of LNM in EGC are the premises to guide the rational choice of clinical treatment.

There are several articles reported on studies of lymph node metastasis in EGC, which fall into two main categories, one being studies based on the SEER database with a large amount of data but lacking data from other institutions for external validation (10, 19). The other category is that of single-center-based studies, which included many study variables but had a small overall sample size and lacked external validation (20–24). The models constructed in the above two types of studies failed to be further validated in terms of clinical generalizability. Our study, with the addition of our center’s data as external validation after this revision, confirmed that the model is still applicable in our center’s cohort, and the addition of the clinical impact curve as an indicator in the method of assessing the model compared with previous studies allowed for a more comprehensive assessment of the model.

In our study, the LNM rate of EGC was 20.84%, which was consistent with the results reported in the previous study by Wang et. al (12), but higher than the 12.3-15.5% in the previous study and 2.5-8.6% in the Japanese scholar’s study (25), which may be due to Japan’s early cancer screening policy and high-grade intraepithelial neoplasia defined as EGC in Japanese diagnostic criteria. Using the population-based SEER database, first, multiple clinicopathological factors associated with an increased risk of LNM were identified by univariate logistic regression: age, tumor size, grade, and ELNC. The study by Lin et al. showed that female gender, tumor diameter >20 mm, submucosal invasion, and undifferentiated tumor histology were independent risk factors with an area under the curve of 0.694 (95% CI: 0.659-0.730) (26). In addition, studies have shown that age, Lauren classification, and lymphatic and perineural invasion are closely related to LNM, and T1b is more prone to LNM than T1a (27, 28). Yin et al. established a first nomogram to identify EGC patients at high risk for LNM using preoperative indicators, the model incorporated 6 independent predictors including tumor size, gross features, histological differentiation, P53, CA19-9, and lymph node status reported by computed tomography, the model has a C-index of 0.82 (95% CI: 0.78-0.86), which has a high clinical value (22), but due to the relatively small number of included studies. Less, the convincing power of the results is limited.

In this study, we found that, as in many previous studies, a nomogram of constructed logistic regression showed that age, tumor size, grade, and ELNC were risk factors for LNM, and tumor location was not associated with LNM, consistent with previous findings (22, 29). The AUC value of the model was 0.698 (95%CI: 0.679-0.717), The correction effect of the calibration was satisfactory and the DCA decision curve analysis showed strong clinical practicability. The pathological type in this study is not a risk factor for LNM, which is still controversial in several current studies (25, 30), which may be related to the difference in the included study population and the number of cases. Lymphovascular invasion and depth of tumor invasion have also been shown to be risk factors for the development of LNM in EGC, possibly due to the abundance of lymphatic vessels in the lamina propria and submucosa (25, 31). Unfortunately, due to the limitations of the SEER database, these two indicators were not included in our study. It is worth mentioning that ELNC was associated with LNM in this study, which may be closely related to the presence of lymphatic micrometastases (32), and examining more NLNCs can improve the detection rate of potentially metastatic-positive lymph nodes (33). Lou et al. explored the significance of lymph node micrometastasis (LNMM) in T1N0 EGC, and LNMM may be a key mechanism of recurrence after surgical treatment in T1N0 EGC patients (34). Therefore, establishing a risk model for predicting LNM can help improve the risk stratification of patients with EGC and improve the accuracy of diagnosis.

In the analysis of the prognosis of EGC patients, the 3-year and 5-year OS of EGC patients were 77.6% and 68.0%, respectively, which is much lower than that of most Japanese studies reporting that the 5-year and 10-year survival rates of EGC patients were both above 90%. In Western studies, 5-year survival rates ranged from 68.0% to 92.0%. The difference in survival may be due to the higher incidence of diffuse histotype in Western countries and less advanced endoscopic procedures, where surgical resection and D2 lymphadenectomy are considered the gold standard of care (18). Multivariate Cox regression results showed that LNM was a significantly poor prognostic factor (HR: 1.786, 95%CI: 1.512-2.111, P<0.001). In addition, age, gender, race, primary site, tumor size, distant metastasis, and whether surgery and ELNC were independent prognostic factors, while radiotherapy, chemotherapy, and pathological type and grade were not associated with prognosis. ELNC is not only a risk factor for LNM but also an independent prognostic factor. The 8th edition of the AJCC guidelines does not clearly define the minimum number of lymph nodes to be dissected in patients with T1 gastric cancer undergoing surgical treatment, but our study shows that the ELNC is more than 11. Lymph nodes showed a better prognosis than ≤11 (HR: 1.786, 95%CI: 1.512-2.111, P<0.001), so for EGC patients undergoing surgical treatment, we recommend that the total number of lymph nodes to be dissected should be at least 11. Sun et al. fit a β-binomial model of the number of lymph nodes to be examined for different primary tumor stages. The study concluded that examining 11 lymph nodes could reduce the probability of missing positive lymph nodes to <10%, which is required for patients with EGC. At least six lymph nodes were dissected (35). Population-based results showed no effect of chemotherapy on prognosis in EGC, but a previous analysis of patients with pT1 GC showed that curative surgery alone is sufficient for patients with pT1N0 and pT1N1. Xelox showed no survival advantage in pT1N2 patients. If adverse reactions are considered, S-1 is the best choice for pT1N2 patients. Xelox is recommended for pT1N3 patients (36). Minerva Chirurgica et al. evaluated the prognostic significance of preoperative serum albumin values and metastatic lymph node ratios in patients with gastric cancer. The results confirm that with albumin, age, resection type, perineural invasion, and ratio of metastatic lymph nodes, T and TNM stages were significant predictors of cancer-specific survival (CSS) (37). In addition, some studies have reported the prognostic value of CD44 Variant 9, Ki-67, and microsatellite instability in EGC, but these conclusions need to be confirmed by more future studies (38, 39).

This study has several limitations. First, this is a retrospective analysis and may be subject to data selection bias. Second, our study lacked serum pepsinogen (PG), serum gastrin-17 (gastrin-17, G-17), Hp infection detection, tumor markers, preoperative imaging, and related ESD or EMR treatment data. Finally, the study population is derived from the SEER database, which mainly reflects the data of Western countries. Although validation of the nomogram with an external cohort may help avoid overfitting of the model, the number of cases in the external validation cohort may have been insufficient. The clinical practices of Eastern and Western countries in the treatment of EGC are very different, so the data of the Eastern population will be required for external validation in the future.

## 5 Conclusions

In summary, we established a nomogram for predicting LNM in EGC patients through logistic regression, and internal validation showed that the model had a good discriminative ability, accuracy, and clinical applicability. Independent prognostic factors were identified by Cox regression, and the results showed that EGC patients had better prognoses when the number of dissected lymph nodes exceeded 11. It is hoped that our results can help clinicians make individualized clinical decisions for EGC patients and facilitate the process of individualized treatment.

## Data availability statement

The original contributions presented in the study are included in the article/supplementary material. Further inquiries can be directed to the corresponding authors.

## Ethics statement

Written informed consent was not obtained from the individual(s) for the publication of any potentially identifiable images or data included in this article.

## Author contributions

JL, TC, and ZH contributed to the study design and wrote the article. YM and WX completed the data analysis. YY and WX generated and improved the figures and tables. KC and HL proofread the manuscript. XC and WW reviewed the article. All authors contributed to the article and approved the submitted version.
